# Pest Control Potential of Social Wasps in Small Farms and Urban Gardens

**DOI:** 10.3390/insects10070192

**Published:** 2019-06-28

**Authors:** Fábio Prezoto, Tatiane Tagliati Maciel, Mateus Detoni, Angie Zuleidi Mayorquin, Bruno Corrêa Barbosa

**Affiliations:** 1 Laboratório de Ecologia Comportamental e Bioacústica, Depto. de Zoologia, Programa de Pós-graduação em Comportamento e Biologia Animal, Universidade Federal de Juiz de Fora, Juiz de Fora 36036-900, Brazil; 2 Department of Zoology, University of Otago, 340 Great King Street, P.O. Box 56, Dunedin 9054, New Zealand

**Keywords:** biological control, artificial shelters, colony management

## Abstract

In environments undergoing constant transformation due to human action, such as deforestation and urbanization, the emergence of pests has become a challenge for agriculture and human welfare. In Brazil, over a thousand tonnes of pesticides are used annually, causing serious environmental damage such as the decline of insect populations. It is necessary to search for control alternatives in order to reduce the environmental impact caused by insecticides. This review aims to describe the use of social wasps as agents of biological control, focusing on the perspectives of their use in small farms and urban gardens, and to discuss the benefits of using this method. Studies have shown that 90–95% of the prey captured by wasps in small crops is made of leaf-eating caterpillars. In urban gardens, wasps diversify their prey, among which potential disease vectors, such as dipterans, stand out. We outline techniques for managing social wasp colonies in small farm and urban garden settings, including the use of artificial shelters. Among the advantages of using wasps as control agents, we highlight the practicality of the method, the low operational cost, the absence of prey resistance and the decrease of the use of insecticides.

## 1. Introduction

Human action transforms natural environments radically through deforestation for the expansion of agricultural and urban areas. In 2008, for the first time in recorded history, more than half of the human population worldwide was reported to live in urban centers. It is estimated that by 2025, this urban proportion might increase to two-thirds of the human population [1].

Continuous environmental transformation has a direct impact on entomological fauna, since only a small number of species are able to survive and thrive in altered ecosystems. Of these, pest species cause losses for the economy and human welfare. In many areas, the main strategy for attempting to keep pest populations under control and minimize losses has been the intensive application of pesticides. In Brazil, for instance, 936,000 tonnes of pesticides have been sold in 2014, costing 6.5 billion US dollars to the farming industry [2].

According to a 2019 publication by Sanches-Bayo and Wyckhuys [3], based on 73 reports on the decline of insect fauna over the world, extinctions are happening at an alarming rate and may reach 40% of certain insect taxa in the next decades. These authors outline environmental changes from deforestation, urbanization and chemical pollution as the main causes for the decline of insect communities. Within chemical pollutions, synthetic insecticides applied on intensive agriculture are highlighted as a cause for special concern [3]. 

This scenario presents an urgent need for insect pest control strategies that reduce environmental impact. The exploitation of natural enemies as agents of biological control stand out as a sustainable alternative. Conservation biological control stands out as the most desirable way to manage pest arthropods. However, due to the complexity of interactions between plant populations, pest arthropods, and natural enemies, this practice still faces challenges to field application [4]. Among the advantages of this control technique when compared to synthetic insecticides, we can highlight lower operational cost, absence of prey resistance and no environmental pollution [5].

In 1951, DeBach [6] was already discussing the importance of social wasps as controllers of countless agricultural pests, especially during population booms when many host-specific natural enemies are inefficient. By 1957 North Carolina, USA, Rabb and Lawson [7] demonstrated that the introduction of *Polistes* to tobacco crops resulted in the reduction of 68% of the damage caused by the caterpillar *Manduca sexta* (Linnaeus, 1763). In China, 1976, experiments carried out by the Institute of Agricultural and Forestry Sciences of Shang-Chiu [8] resulted in the control of 70–80% of *Helicoverpa armigera* (Hübner, 1805) and *Etiella zinckenella* (Treitschke, 1832) within only 5–7 days after the introduction of colonies of *Polistes* wasps in a cotton farm.

Although social wasps are abundant in a diversity of environments throughout the globe (Stenogastrinae with 53 spp, Vespinae with 67 spp and Polistinae with 943 spp), few investigations have focused on their potential as biological pest control agents [9,10,11]. This communication aims to provide insights on the potential use of social wasps as agents of biological pest control in small farms and urban gardens. We also discuss the benefits of this method as a sustainable, low cost alternative for pest control.

## 2. Foraging Activity in Social Wasps

The foraging activity of social wasps includes the search and attainment of essential resources for the construction and maintenance of their colonies. Through this behavior, ecological interactions are formed such as predation and pollination. For most species of wasps, increased temperatures combined with high luminosity and low air humidity are factors that stimulate colony foraging activity [12]. Although foraging usually occurs through the whole day, it is most intense during the warmest hours of the day (e.g., from 11 a.m. to 3 p.m.) [13]. Different from solitary species, social wasps continuously search for food resources, which requires a daily foraging effort throughout the colony’s life cycle.

Food resources collected by social wasps can be divided into carbohydrates and proteins. Carbohydrates are used mainly to feed adults and are obtained from honeydew [14], ripe fruit [15], and both floral and extrafloral nectaries [16]. Foragers may participate in the pollination of flowers through these ecological interactions. Besides sugary substances, colonies of wasps need great amounts of protein, which are used to feed their larvae [17]. Various studies report a tendency for wasps to prey on Lepidoptera larvae (caterpillars), although other insect taxa are also captured [12,18]. It is through this prey capture that wasps can control pest populations in small farms and urban gardens. For instance, it is estimated that a single colony of *Polistes* paper wasps capture over 4 thousand items of prey throughout its life cycle [19].

Wasp foragers look for resources in a species-specific radius of 100–300 m around their nests [19], which determines their flight range. Knowing the flight range of a species is helpful for planning the ideal spacing between colonies during management efforts to avoid overlap in foraging ranges.

## 3. Prey Captured by Social Wasps

**Figure 1:**
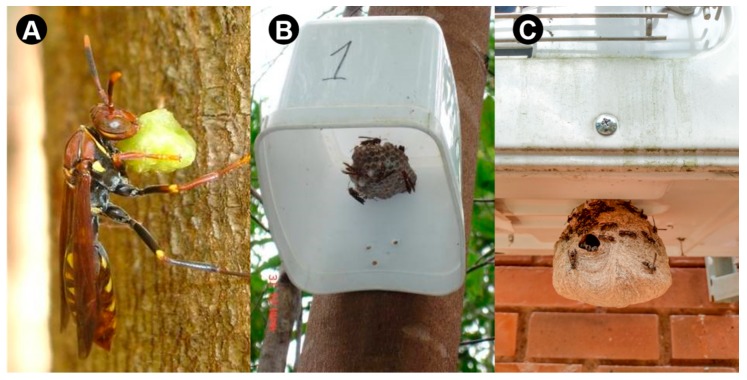
(**A**) Social wasp holding a fragmented caterpillar prey in a small farm environment. (**B**) Example of artificial shelter used for transferring colonies of social wasps during their management for pest control purpose in small farms; inside of the shelter is highlighted in the image. (**C**) A well-established colony of social wasps in an urban area.

The successful capture of prey is directly reflected in the development of the colony and in the production of new workers, which highlights the value of animal protein on the wasps’ diet. Foragers locate their prey using chemical cues, especially through the detection of allelochemicals produced by plants during herbivory [20], and through visual orientation via prey movement [12]. Social wasp prey identification reveals that, although generalist, wasps tend to capture soft-bodied terrestrial arthropods [14,21] (Figure 1A). Leaf-eating caterpillars (Lepidoptera) are the most common choice, making up 90–95% of the captured prey [22,23,24,25,26,27,28,29,30,31]. Other common preys include immature and adult insects such as Diptera, Dermaptera, Orthoptera, Odonata, Hemiptera, Coleoptera, and Hymenoptera, but also spiders [14,21,32]. The preference for preying upon Lepidoptera is a good indicator that wasps could act as biological control agents for economically destructive caterpillar populations such as *Spodoptera frugiperda* (Smith, 1797), *Chlosyne lacinia saundersii* Doubl. & Hew, 1849, *Alabama argillacea* (Hübner, 1818), *Anticarsia gemmatalis* Hueb., 1818 and *Heliothis virescens* (Fabricius, 1781), which make up the most common pests abundantly found in small farms in Neotropical environments such as Brazil [5,22].

**Figure 2:**
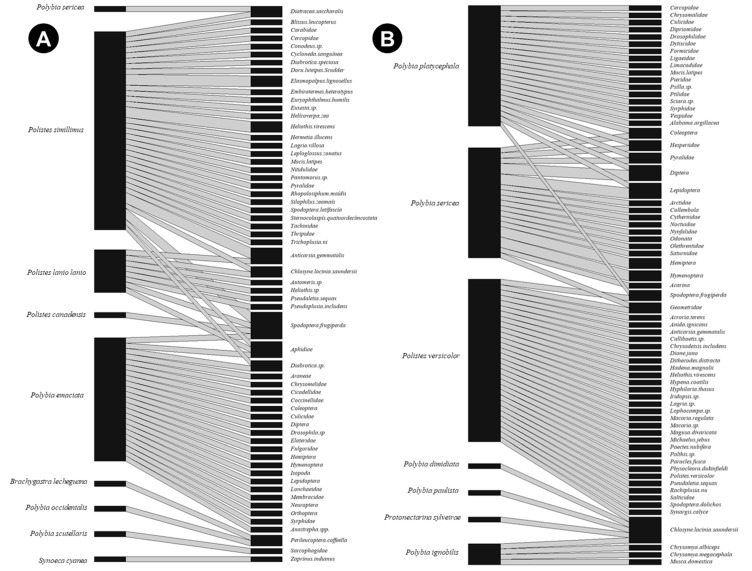
Ecological network between social wasps and their prey in small farms (**A**) and urban gardens (**B**). The left columns on each side represent social wasps, while the right columns represent captured prey. The height of each rectangle is directly proportional to the number of records made for the species, while links represent the number of records made between the pair of species.

Studies investigating the protein foraging effort in social wasps have led to the discovery of two key behavioral traits: generalism and opportunism [12]. The opportunistic–generalist foraging behavior allows wasps to survive in a diversity of environments, including altered ecosystems such as small farms and urban gardens, by allowing them a wide and convenient choice of prey [12]. Based on records made in the prey identification studies cited on this paper, we built two ecological networks: one for small farms [24,33,34,35,36,37,38,39,40] and another for urban gardens [41,42,43,44] (Figure 2A,B). This diagram (Figure 2) was built from a binary matrix using “social wasp species” and “taxa captured by social wasp foragers”. The graph was generated by using the package *bipartite* [45] in the R software [46].

In those networks, wasps prey on Lepidoptera in higher proportions at small farms, probably due to the higher abundance of leaf-eating insects in farming environments. Greater diversity of captured prey is found in urban gardens, with Diptera more captured than Lepidoptera. This versatility in social wasp diet composition is evidence of their foraging behavioral plasticity. In addition, it is important to note that Diptera includes important disease vectors such as the mosquitoes of the Culicidae family, especially the *Aedes* genus. Our analysis suggests that social wasps could be valuable biological control agents, standing out due to their wide prey selection and absence of the typical setbacks seen in chemical control such as the creation of resistance in pest populations [47].

## 4. The Management of Social Wasp Colonies for Exploitation as Pest Predators

The critical step towards exploiting ecological services provided by social wasps relies on the ability to handle wasp colonies and manage their populations. Ubiquitous human impact on the environment often results in very high pest densities [48]. Even if a diverse community of social wasps is naturally present, it may be unable to contribute to significant pest control due to its own behavioral and ecological thresholds [12,49]. Therefore, the exploitation of social wasps as pest predators requires methods that allow for artificially increasing their own population densities in the site of interest.

The rearing of social insect colonies in artificial environments for economical purposes is best represented by apiculture and the methods designed for allowing beehives to thrive in semi-natural or artificial systems [50]. However, despite taxonomical relatedness and behavioral similarities between honey bees and social wasps, the rearing of wasp colonies in systems with any degree of artificiality is considerably more challenging. Social wasp colonies are sensitive to manipulation, and while individuals survive for considerable periods in laboratory settings [51], there no reports of a social wasp colony retaining its normal behavior or successfully reproducing in the lab. The European paper wasp, *Polistes dominula* (Christ, 1971), is a clear exception, but even with the possibility of reproduction, their behavior in artificial environments seems to be strongly stereotyped [52].

With the rearing of many social wasps infeasible, researchers have turned to the alternative solution of transferring colonies between habitats using artificial shelters allows for the creation of conveniently placed and sized populations. This is an important method for researching their biology and behavior, but may also be used to exploit pest predation in agriculture [33]. However, this alternative also presents limitations: wasps are prone to abandon their nests after the relocation, likely a response to the stress of manipulation or an unsuitable surrounding environment—such as inadequate sheltering or lack of environmental resources [32].

Reports of *Polistes* wasp colonies being successfully relocated focused on their potential for pest control [31,33,53,54,55]. *Polistes* nests consist in a single vegetal fiber exposed comb connected to the substrate through a peduncle [56]. Nests are often found on natural or man-made structures that provide shelter against harsh weather conditions, such as tree branches or eaves of buildings [19]. This nest architecture is more helpful in the removal of entire colonies for the transference when compared to the bigger and more structurally complex nests of the swarm-founding Polistinae or the Vespinae.

Relocation methods focus on removing the whole nest from the original substrate and fixating it to an artificial shelter in the new habitat. The removal of the nest from the original habitat is usually carried out at night, to ensure that foragers are in the colony and avoid population loss [33]. The nest and wasp population are kept inside a plastic bag and immediately transferred to the new location, where the nest may be directly glued to the shelter. The bag containing the wasps is then fixed around the shelter overnight to stimulate contact between wasps and the nest and avoid immediate abandonment due to manipulation stress [33]. The shelters consist of thin plastic or wooden boxes without their bottom surface, supported by a vertical wooden beam whose height can be adjusted accordingly to the species nesting habits or research purpose [31]. Nests are placed inside the box, which provides shelter from rain or strong winds, and allows them to forage at will (Figure 1B). The first week after transference is considered critical for the acceptance of the new environment [31], after which the remaining colonies usually show normal activity and development. These methods are noteworthy for their low cost, simple design and considerable success rate (reported to range from 60–75%) [33,55].

For Vespinae wasps, notably in the *Vespula* genus, the adaptation of methods designed to shelter honey bee hives in layered wooden boxes have been successful for research purpose e.g., References [57,58,59]. Underground nests are anesthetized and excavated from their natural habitat, after which their envelope is removed and their combs are individually glued to one or more layers of the artificial ‘hive’ [57]. As with the polistines, the replacement of the hived colonies is carried out at night to minimize population losses [59]. Despite the highly artificial setting, wasp colonies are tolerant to this manipulation and may continue to develop after relocation [59]. However, transferring *Vespula* colonies to a new location is a more delicate process than what has been done with *Polistes* since it involves considerably larger populations, the use of anesthetics, and the ‘hive’ structure is more complex and costly than artificial shelters for paper wasps. This may be a limiting element for the management of *Vespula* for economic exploitation. Nevertheless, their large populations and considerable foraging range (up to more than 300 m from the nest in some species) [60] suggest that even a relocation of a few wasp colonies could have a significant impact on pest populations, especially in areas where other social wasp taxa are underrepresented.

Another limiting aspect of this management technique is the invasive potential of social wasps. As with any other biological control effort, the areas where pest control is desired must undergo careful ecological assessment to avoid the risk of accidental introduction of exotic species to natural environments. Invasive populations of social wasps can cause severe environmental imbalance by becoming pests themselves, when their predation behavior endangers native species [61]. The most obvious example of this risk is the introduction of exotic *Vespula* wasps in the beech forests of New Zealand’s South Island where the colonies’ natural cycle may be disrupted, resulting in very high population densities of wasps which outcompete endangered bird species for food and can prey on native invertebrates to their disappearance [62]. The ideal candidates for colony transference are thus native species with a good natural abundance, minimizing problems caused by increased densities or by handling species with small populations.

## 5. Prey Captured and Used by Social Wasps in Small Farms: A Study with *Polistes simillimus*

Despite what is known about prey captured by social wasps, there is a lack of studies showing social wasps being used in pest control programs. In 1999, research in Brazil by Prezoto and Machado [33] assessed the predatory behavior of *Polistes simillimus* (Zikan, 1951) on the fall armyworm, *S. frugiperda*, in a small corn farm from planting to harvest. To do this, the authors transferred colonies of *P. simillimus* to experimentally design wooden shelters installed around the crop.

In the initial stages, 500 m^2^ of crop was infested with first-instar *S. frugiperda* caterpillars, followed by the transference of 20 *P. simillimus* colonies to the shelters. For 12 weeks, the authors collected prey captured by wasps by intercepting foragers returning to their nests. Around 90% of the prey captured by the wasps was made of Lepidoptera caterpillars, and, of those, *S. frugiperda* was the most common item (23.07%).

By the end of the crop cycle, the population of *S. frugiperda* was reduced by 77.16%. Incidentally, a naturally present second pest species, the American cotton bollworm [*Helicoverpa zea* (Boddie, 1850)], which feeds on corn cobs, had its population reduced by 80% at the same time. The experimental crop had its productivity increased by 15.94% when compared to the control crop, and the average ear mass was 13.07% higher in the wasp-treated crop. These results show that *P. simillimus* was efficient in locating and capturing *S. frugiperda* on the developing ears, where chemical compounds are usually less effective. 

Comparatively, in a study with a similar approach, the social wasp *Polybia ignobilis* (Haliday, 1836) was shown to reduce 70% of the population of *Ascia monuste* (Godard, 1919) caterpillars (Lepidoptera: Pieridae) on kale crops [63]. These results are similar to Prezoto and Machado [32] in which both show a high rate of success in controlling specific pest populations, attesting to the social wasps’ potential as a biological control agent.

## 6. Prey Captured and Used by Social Wasps in Urban Gardens: A Study with *Polybia platycephala*

In a study carried out by Prezoto et al. in 2005 [42], prey captured by the swarm-founding wasp *Polybia platycephala* (Richards, 1978) was analyzed in the Juiz de Fora municipality (21°46′ S 43°21′ W, altitude 800 m) in the southeast of Brazil. *Polybia platycephala* foragers returning to their nests in urban garden areas were intercepted during the warmer periods of the day (10:30 a.m. to 2:30 p.m.) (Figure 1C) and their prey was collected for identification over 70 h of fieldwork. Of the 84 prey items collected from wasps, five insect orders could be identified: Diptera (33.4%), Lepidoptera (28.6%); Hemiptera (12.0%); Hymenoptera (9.4%); and Coleoptera (7.2%).

These results differ from most published research on prey capture for social wasps, which usually show a much higher proportion of Lepidoptera caterpillars (90–95%) [24,26,27]. However, that discrepancy further points to behavioral plasticity in wasp foraging.

The diversity of preys captured by *P. platycephala* in urban gardens is associated with pests present in urban environments, like mosquito and fly larvae, leaf-eating caterpillars, aphids and ant reproductives. Among the Diptera captured by wasps, most individuals belonged to the Culicidae family (26.2%). The fact that Culicidae was well represented on the wasp’s diet is interesting, given that this family includes mosquitoes of the genus *Aedes*, vectors of diseases such as the dengue, zika and chikungunya fevers—all of which are responsible for a number of deaths in Brazil [64]. The results suggest that adequate management of *P. platycephala* colonies in urban areas may play a role in urban pest control efforts, reducing the costs and setbacks of traditional control methods and achieving a balance between prey and predator populations in these environments.

## 7. The Potential of Social Wasps as Biological Control Agents

Social wasps are present in almost every terrestrial biome, with the exceptions being polar regions and high altitudes. About 1000 known species exist, many of which are easily found in urban environments. Among the most common genera we can highlight for pest control include *Polistes*, *Mischocyttarus*, *Polybia, Protopolybia*, *Vespula*, and *Ropalydia* [9,10,11]. Despite their common presence in urban areas, the foraging activity and predation behavior is well known for less than 20 species of social wasps. Part of this knowledge gap could be related to the popular association of wasps to the risk of getting stung, which demotivates studies focused on the group [65]. Still, there are a number of publications that support the use of social wasps as biological control agents [32,53,63,66,67].

Social wasp colony management in artificial shelters is a feasible, simple, and cost-effective technique. This method is better suited to systems such as small farms and urban gardens, since wasps require some environmental complexity in order to maintain their colonies, such as sources of water, access to both sugars and protein, and additional nesting sites. Social wasp biocontrol would therefore have very limited performance in areas such as plantations because of monocrop landscape heterogeneity.

## 8. Conclusions

The predatory behavior of social wasp is beneficial to human populations, since it embodies a readily available, consistent ecological service. Through their ecology and behavior, social wasps represent a potentially sustainable alternative to traditional pest control strategies. Once transferred to a new location, wasp populations may remain active for years by reproducing on their own, thus ensuring a much desired long-term predatory action.
